# Phenotyping the Chilling and Freezing Responses of Young Microspore Stage Wheat Spikes Using Targeted Metabolome and Lipidome Profiling

**DOI:** 10.3390/cells9051309

**Published:** 2020-05-25

**Authors:** Bo Eng Cheong, Olive Onyemaobi, William Wing Ho Ho, Thomas Ben Biddulph, Thusitha W. T. Rupasinghe, Ute Roessner, Rudy Dolferus

**Affiliations:** 1 School of BioSciences, The University of Melbourne, Melbourne, VIC 3010, Australia; cheongb@student.unimelb.edu.au (B.E.C.); whho@unimelb.edu.au (W.W.H.H.); tru@unimelb.edu.au (T.W.T.R.); u.roessner@unimelb.edu.au (U.R.); 2 CSIRO Agriculture & Food, GPO Box 1700, Canberra, ACT 2601, Australia; olive.onyemaobi@csiro.au; 3 Department of Primary Industries and Regional Development, 3 Baron Hay Court, South Perth, WA 6151, Australia; ben.biddulph@dpird.wa.gov.au

**Keywords:** wheat, spike, cold tolerance, phenotyping, reproductive development, metabolomics, lipidomics

## Abstract

Chilling and frost conditions impose major yield restraints to wheat crops in Australia and other temperate climate regions. Unpredictability and variability of field frost events are major impediments for cold tolerance breeding. Metabolome and lipidome profiling were used to compare the cold response in spikes of cold-tolerant Young and sensitive variety Wyalkatchem at the young microspore (YM) stage of pollen development. We aimed to identify metabolite markers that can reliably distinguish cold-tolerant and sensitive wheat varieties for future cold-tolerance phenotyping applications. We scored changes in spike metabolites and lipids for both varieties during cold acclimation after initial and prolonged exposure to combined chilling and freezing cycles (1 and 4 days, respectively) using controlled environment conditions. The two contrasting wheat varieties showed qualitative and quantitative differences in primary metabolites involved in osmoprotection, but differences in lipid accumulation most distinctively separated the cold response of the two wheat lines. These results resemble what we previously observed in flag leaves of the same two wheat varieties. The fact that this response occurs in tissue types with very different functions indicates that chilling and freezing tolerance in these wheat lines is associated with re-modelling of membrane lipid composition to maintain membrane fluidity.

## 1. Introduction

Frost events can cause major yield losses to cereal crops in many temperate climate regions in the world. Wheat crops in Australia are grown during winter and they flower in early spring to avoid the hot summers and to take advantage of available soil moisture. The sensitive stage of flowering is therefore frequently exposed to frost. The annual yield loss due to frost events to the Australian grains industry is estimated to be A$360 million [1,2,3,4,5]. The problem is exacerbated by climate change. In China and Australia, spring frosts have become increasingly frequent since the 1960s and the length of the frost season has been extended by one month, leading to more frequent yield losses due to frost damage [5,6,7,8].

In field conditions, severity, occurrence, and timing of frosts during plant development are highly variable, making screening and selection for wheat germplasm with higher frost tolerance very difficult. Using controlled environment experiments is complicated by the fact that radiative frost conditions are difficult to simulate [9,10,11,12]. Phenology and flowering time play an important role in avoiding exposure of the sensitive reproductive structures, anthers in particular, to frosts [5,7,13,14]. The increased frequency of frost occurrences in field environments indicates the need for wheat germplasm with improved tolerance to frost conditions.

Acclimation to non-freezing chilling or cold conditions (above zero degrees, low temperatures) plays an important role in establishing tolerance to freezing (below zero degree) temperatures. Frosts occur when ice crystallization takes place in plant tissues due to a combination of freezing temperatures, humidity, and temperatures falling below the dew point [4,9]. Plants respond during the chilling period by mounting an acclimation response to protect macromolecules and cellular functions against reactive oxygen species (ROS) and to protect tissues against ensuing freezing and frost conditions [15,16,17,18]. Low temperatures were shown to lead to accumulation of osmolytes (various sugars, polyols, betaines, amines, and amino acids) that may act as cryo-protectants to protect the cells against freezing and associated water loss [19,20,21,22,23]. Importantly, protection of membrane fluidity through changes in unsaturated lipid levels in the membrane was also found to play an important role in protecting plant tissues against frost damage [24,25,26]. However, it remains unclear how quantitative and qualitative differences in the accumulation of these compounds during the acclimation response correlates with differences in frost tolerance levels and information about physiological differences in cold-acclimation responses between cold-tolerant and sensitive germplasm is also missing.

Changes in metabolite composition provide a powerful tool to explore cold acclimation and potentially tolerance to chilling and frost conditions. Metabolomics is therefore a technology that can contribute to more accurate and reliable phenotyping of physiological changes in plant tissues [27,28,29]. Metabolite and lipidome analyses in wheat flag leaves revealed significant differences in the cold response to chilling of frost-tolerant and sensitive wheat lines: membrane fluidity was maintained mainly by increasing unsaturated lipid levels in cold-tolerant wheat. This response was absent in cold-sensitive wheat, where a water stress-type response was observed instead [30].

The aim of this study was to use metabolomics and lipidomics profiling to investigate changes in cold acclimation in the reproductive tissues (spikes) of two wheat varieties with contrasting cold tolerance. Herein we used the same wheat varieties with our previously published paper, which are the cold-tolerant Young and cold-sensitive Wyalkatchem [30]. Metabolites that can reliably differentiate the response of a cold-tolerant and sensitive wheat line are useful as “diagnostic markers” in wheat cold-tolerance phenotyping. To achieve this, we will have to test the reliability of candidate metabolite markers identified in this study first on a wider range of wheat chilling/freezing tolerant and sensitive germplasm before using these markers in routine phenotyping tasks. We scored metabolite and lipidome changes during cold acclimation after initial exposure (1 day) and prolonged exposure to cold (4 days) in order to identify diagnostic metabolite markers that could reliably distinguish the cold-tolerant wheat variety from the cold-sensitive one. We then compared the cold acclimation response between spikes and flag leaves for the two wheat varieties to identify differences in cold acclimation and to determine which tissue is most suitable for cold tolerance phenotyping. Wheat spikes are physiologically different from flag leaves in that the former are actively developing sink tissues, while the latter are static photosynthetic source tissues. Comparison of the primary metabolite and lipid changes in response to cold for both wheat varieties will enable us to distinguish which metabolites are critically important for cold acclimation.

Abiotic stresses such as cold affect sink–source relationships and distribution of sugars in the plant [31]. Source and sink tissues may therefore react differently to cold and reveal accumulation of different metabolites during cold acclimation. At the young microspore (YM) stage and during grain-filling, the reproductive structures in cereals represent the strongest sink strength of the plant. This is essential to attract sugars for reproductive development and grain filling. Sink strength is highest during pollen formation after meiosis and during grain development after fertilization [32,33,34,35,36,37]. Abiotic stresses such as cold and drought reduce sink strength in anthers of stress-sensitive lines, while stress tolerance was correlated with a higher potential to maintain sink strength [38,39]. In this study we demonstrate that cold acclimation in spikes and flag leaves shows some similarities in terms of accumulation of compounds involved in osmoprotection and defense against reactive oxygen species (ROS). However, more importantly, both tissues share a similar mechanism in modifying their membrane lipid composition in response to cold. These findings highlight the importance of preserving membrane fluidity and functionality in different plant tissues during cold acclimation in wheat. Metabolomics and lipidomics can be exploited as a phenotyping strategy to discriminate cold-tolerant from cold-sensitive wheat germplasm.

## 2. Materials and Methods

### 2.1. Controlled Environment Wheat Growth, Cold Treatment, and Tissue Sampling

**Figure 1:**
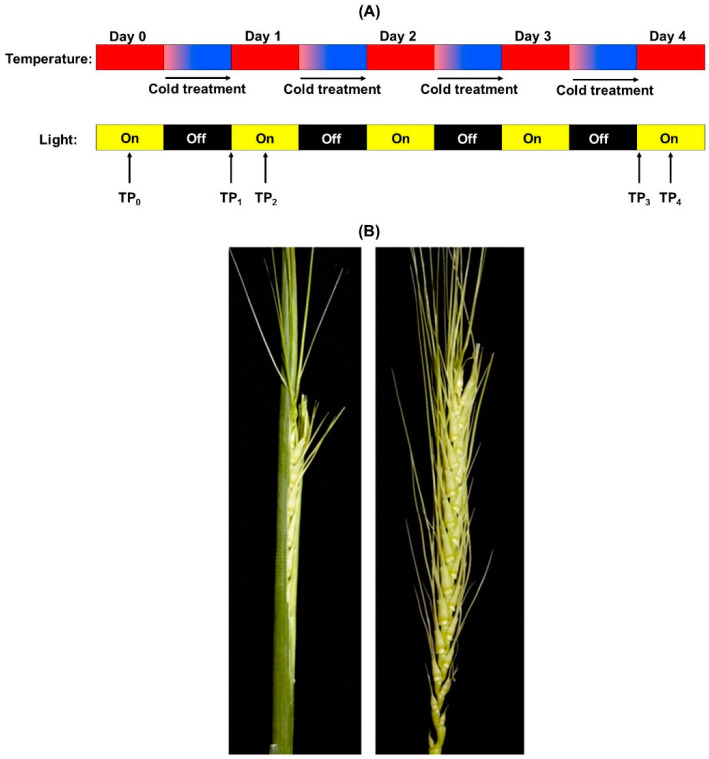
(**A**) Schematic diagram showing the controlled environment design of the cold treatments and lighting conditions (see Section 2.1). Yellow bars show day-light conditions, black bars dark conditions. Arrows labelled TPx indicate where samples were harvested from wheat plants. (**B**) Pictures showing the morphological stage of the young microspore (YM) stage wheat spikes used in this study. At the YM stage, wheat spikes have been shielded from light exposure by the surrounding leaf sheaths (left picture). The picture on the right shows the dissected spike used for metabolite measurements.

Wheat varieties Wyalkatchem (cold-sensitive) and Young (cold-tolerant), plant growth, and cold treatment conditions were as previously described [30]. The two wheat varieties we used were chosen based on their performance in field chilling and frost trials and can be differentiated in controlled environment assays based on spike sterility levels after a four-day chilling/freezing treatment [30]. Plants were grown in the glasshouse under natural lighting conditions and controlled temperature regime (24/16 °C light/dark). When plants reached the YM stage of pollen development, the YM-stage tillers were tagged and plants were transferred to a Conviron PGC 20 growth chamber (Conviron, Winnipeg, Canada) with the following settings (Figure 1A): 12 h at 21 °C (light period), 4 h linear cooling gradient in the dark to −3 °C, followed by 8 h in the dark at constant −3 °C (12/12 light/dark cycle, using 400 μmol.m^−2^.s^−1^ light intensity) as a cold cycle. The cold-treatment uses a simplified profile of a typical mid-August early morning chilling/frost event in the Southern Australian wheat growing areas. The linear temperature decline in the profile allows induction of an acclimation response before a period of freezing temperatures. Despite the use of below-zero temperatures, this treatment does not cause ice crystal formation. Ice crystallization depends on humidity and dew point which is hard to control in controlled environments. Super-cooling above the canopy [9] or treatments with ice-nucleating agents can cause ice crystallization but were avoided because of secondary effects and because ice formation is not essential for this study that focuses on varietal differences in acclimation to chilling conditions. YM-stage spike samples were harvested at the start of the treatment (TP_0_, TP = time point), at the end of the first overnight cold cycle (TP_1_), and 6 h into the light cycle after the first cold treatment (TP_2_). Samples TP_3_ and TP_4_ are equivalent to TP_1_ and TP_2_, but samples were harvested after the fourth consecutive cold treatment to study the effect of long-term cold treatment on the acclimation response [30] (Figure 1A). Each sample used for metabolite measurements consisted of three spikes, harvested from different plants, and for each time point we collected three biological repeat samples for both the cold-tolerant and sensitive wheat varieties.

### 2.2. Analysis of Sugars, Organic Acids, Amino Acids, and Amines

All chemicals and solvents for metabolite measurements were purchased from Sigma-Aldrich (Castle Hill, NSW, Australia) and were of analytical or mass spectrometry grades. For sugars, organic acids, and amines, tissue extraction was performed as previously described [30,40], with some modifications. Aliquots of 20 mg of frozen spike material were weighed in Cryomill tubes (Precellys 24, Bertin Technologies, Rockville, MD, USA). To each sample, 500 µL of methanol containing 4% of internal standards (from a stock solution containing 0.5 mg/mL of ^12^C_6_-sorbitol and 0.5 mg/mL ^13^C5-^15^N-L-valine) was added, followed by vortexing for 30 s and homogenization at −10 °C using a Cryomill (3 × 45 s at 6100 rpm). The samples were then extracted for 15 min at 30 °C in a thermomixer at 850 rpm, and subsequently centrifuged for 15 min at 4 °C at 13,000 rpm. The supernatants were combined into new tubes and 500 µL of 0.2% formic acid (in water) was added into the Cryomill tubes containing the previously ground tissue pellet. The samples were vortexed for 30 s, and spun at 13,000 rpm for 15 min at 4 °C. The supernatants were then combined with the methanolic extracts from the previous centrifugation and stored at −80 °C for subsequent analyses.

For the analyses of sugars and organic acids, an 80 µL aliquot of the supernatants was transferred into new glass vial inserts and dried in vacuo without heating. Prior to the GC-QqQ-MS analysis, the dried extracts were derivatized with methoxyamine hydrochloride in pyridine and bis-(trimethylsilyl)-trifluoroacetamide (BFTFA) as previously described [40]. Briefly, samples were re-dissolved in 20 µL of 30 mg/mL methoxyamine hydrochloride in pyridine and derivatized at 37 °C for 120 min with mixing at 500 rpm. After addition of 20 µL N,O-bis-(trimethylsilyl)-trifluoroacetamide (BSTFA), the samples were incubated for 30 min with mixing at 500 rpm at 37 °C. Derivatized samples were allowed to equilibrate for 60 min and 1 µL aliquots of each sample were injected into a GC-QqQ-MS system, consisting of a Gerstel 2.5.2 Autosampler, a 7890A Agilent gas chromatograph, and a 7000 Agilent triple-quadruple MS (Agilent Technologies, Santa Clara, CA, USA) with an electron impact (EI) ion source. The instrument settings were the same as described previously [30,40]. For calibration and quantification of sugars and organic acids in the flag leaf samples, we used serial concentrations of calibration standards (Table S1). Standards included 24 sugars (sugars, sugar phosphates, sugar acids, and sugar alcohols) and 19 organic acids, derivatized and subjected to GC-QqQ-MS analysis as described previously [30,40].

For the amino acid and amine analyses with LC-QqQ-MS, 10 µL of supernatants were transferred into new glass vial inserts. The amino acids and amines in the supernatants were then derivatized with 6-aminoquinolyl-N-hydrosysuccinimidyl carbamate (AQC) reagent [30,40,41]. Briefly, 10 µL samples were added to 70 µL of borate buffer (200 mM, pH 8.8 at 25 °C) containing 10 mM TCEP, 1 mM ascorbic acid, and 50 µM 2-aminobutyrate. The resulting solution was vortexed before adding 20 µL of 6-aminoquinolyl-N-hydrosysuccinimidyl carbamate (AQC) reagent (200 mM dissolved in 100% acetonitrile, ACN) and then vortexed immediately. The samples were heated with shaking at 55 °C for 10 min and centrifuged at 13,000 rpm at room temperature and then transferred to HPLC vials containing inserts (Agilent, spring-less glass inserts, 250 µL) prior to injection. Aliquots (1 µL) of the derivatized samples were immediately injected into a LC-QqQ-MS system comprising of an Agilent 1200 LC-system coupled to an Agilent 6410 Electrospray Ionization-Triple Quadruple-MS. The settings of the LC-MS instrument were previously described [30,40]. For calibration and quantification of amino acids and amines in the flag leaf samples, a series of concentrations of calibration authentic standards comprising of 29 amino acids and amines (Table S1), mixed with a sulfur-containing compound solution, were prepared, derivatized, and subjected to LC-QqQ-MS analysis same as previously described [30,40].

### 2.3. Analysis of Lipids

The extraction of lipids was carried out as described previously [30,42], with modifications. Briefly, 20 mg freshly frozen spike samples were homogenized in 500 µL of a 2:1 methanol:chloroform mixture using a Cryomill (Precellys 24, Bertin Technologies, Rockville, MD, USA) for 3 × 45 s at 6100 rpm (−10 °C). The extracts were shaken at 750 rpm for 15 min at 30 °C and centrifuged at 13,000 rpm for 15 min. The supernatants were transferred to new tubes, while 500 µL of a 2:1 methanol:chloroform mixture was added again to the remaining residue. The tubes were vortexed and centrifuged again at 13,000 rpm for 15 min. The supernatants were combined and dried down in vacuo and then re-constituted in 100 µL of 1:1 butanol:methanol for the subsequent LC-QqQ-MS analysis using an Agilent 1200 LC-system coupled to an Agilent 6410 Electrospray Ionization-Triple Quadruple-MS system. An injection of 5 µL of each total lipid extract was chromatographically separated on an Ascentis Express RP-Amide 50 × 2.1 mm, 2.7 µm HPLC column (Sigma-Aldrich, Castle Hill, NSW, Australia) using an 8 min gradient from 0% A to 100% B, which was then held for 2 min and followed by a 4 min column re-equilibration with a flow rate of 0.18 mL/min. The mobile phases were A: 10 mM ammonium formate in water:methanol:tetrahydrofuran (50:20:30, v:v:v); B: 10 mM ammonium formate in water:methanol:tetrahydrofuran (5:20:75, v:v:v). Lipid species were identified and quantified based on multiple reaction monitoring (MRMs) established using external lipid standards and references from the LIPIDMATCH database (https://github.com/GarrettLab-UF/LipidMatch/releases/tag/v2.0.2) as listed in Table S2, with a 5–20 ms dwell time for the simultaneous measurements of up to 100 compounds. We used optimized parameters for capillary (4000 V), fragmentor (60–160 V), and collision voltages (20–40 V). In all cases, the collision gas was nitrogen with a flow rate of 7 L/min. The external lipid standards used were lysophosphatidylcholine LPC (17:0), lysophophatidylglycerol LPG (16:0), phospatidylcholine PC (20:0) and PC (34:1), phosphatidylethanolamine PE (34:0), phosphatidylglycerol PG (34:1) and PG (36:0), phosphatidylinositol PI (34:2) and PI (36:2), and phosphatidylserine PS (34:0) (Table S2). Detected lipid species were annotated by lipid class designation, followed by the “total number of carbon atoms in the fatty acid chains” and the “total number of double bonds in the fatty acid chains”, separated by a colon sign.

### 2.4. Processing of Raw Data

Raw metabolite and lipid data were processed and analyzed using Quantitative Analysis MassHunter Workstation software for QQQ (Agilent Technologies, Santa Clara, CA, USA). The level of identification was carried out based on the Metabolomics Standards Initiative (MSI) requirements [43]. For all measured metabolites (sugars, organic acids, amino acids, and amines), absolute concentrations were determined for all the metabolites except for fructose-6-phosphate and glucose-6-phophate as the linear standard curves for these two metabolites could not be generated for quantification due to very high abundance of these two metabolites in the spike samples. The concentration unit for all the metabolites was expressed as picomole/mg of fresh weight except responses normalized to mg of fresh weight for fructose-6-phosphate and glucose-6-phophate (Table S3). All the metabolites measured at MSI Level 1, as the identification was based on multiple reaction monitoring (MRMs) established using authentic standards (Table S1). For lipids, responses were normalized to mg of fresh weight, FW (Table S4). Although single authentic lipid species were used for each of the phospholipid class, the identification of individual lipid species was based on both the MRM experiment and retention time and was therefore measured at MSI Level 2 (Table S2).

### 2.5. Statistical Analysis of Metabolite and Lipid Data

The statistical analysis for metabolite and lipid data was performed by using MetaboAnalyst v3.0 [44]. For normalization of metabolite data which are expressed as absolute quantity (picomole/mg of fresh weight), the data was log_2_-transformed and mean-centered. For normalization of lipid data which in response/mg of fresh weight, the data was first normalized by median, log_2_-transformed, mean-centered, and divided by the standard deviation of each variable. Next, for the statistical significance determination of metabolites and lipids in each variety or between varieties, the normalized data was subjected to one-way analysis of variance (ANOVA), with a false discovery rate (FDR)-adjusted *p*-value of 0.05 as significance level [45]. This was followed by the Tukey’s honestly significant difference (HSD) post-hoc test to perform significant pairwise comparisons using MetaboAnalyst 3.0 or Graphpad Prism 7.0 (GraphPad Software, La Jolla California, CA, USA). In order to determine which metabolites or lipids responded specifically to short cold stress (TP_1_), prolonged cold stress (TP_3_), diurnal fluctuations when the spikes were exposed to normal day-time temperature of 21 °C for 6 h (TP_2_ and TP_4_), we performed pairwise comparisons of the selected two groups: TP_1_ vs. TP_0_ (for short cold stress), TP_3_ vs. TP_1_ (for prolonged cold stress), TP_2_ vs. TP_1_, and TP_4_ vs. TP_3_ (for recovery and the effect of diurnal fluctuations on metabolite levels) in each variety, and also comparisons at every time point between the two varieties (varietal differences) via Student’s *t*-test using MetaboAnalyst and GraphPad Prism software. The Student’s *t*-test for comparing the unsaturation to saturation lipid ratio (using the response/mg values) between Wyalkatchem and Young at different time points was performed in Microsoft-Excel. All plots shown in the results section were generated either using GraphPad Prism 7.0 software, MetaboAnalyst 3.0 software, and the Venn diagram webtool (http://bioinformatics.psb.ugent.be/webtools/Venn/).

## 3. Results

### 3.1. Metabolite and Lipid Profiling Using YM Stage Wheat Spikes

The spike tissues analyzed in this study were collected from the same plant material treated by the same controlled environment cold treatment as the previously analyzed flag leaf samples [30]. YM stage spikes are actively developing sink tissues inside the leaf sheaths of the wheat plant where they lack direct exposure to light (Figure 1B). This material needs to be destructively harvested (i.e., plants will not produce seeds) for metabolite analysis. The cold-tolerant wheat variety Young used in this study consistently performs better than the cold-sensitive variety Wyalkatchem in terms of spike grain yield in both field trials of the Australian National Frost Program (ANFP) and our controlled environment assays [30]. The aim of the time-course experiment was to capture changes in cold acclimation after a single night of exposure to cold (TP_1_,_2_) and after a prolonged 4-day treatment (TP_3_,_4_). To be able to measure the effect of daytime recovery and circadian rhythms, we also compared metabolite levels in samples harvested in the morning immediately after overnight cold treatment (TP_1_,_3_) to the samples harvested after 6 h of day-time recovery at normal temperatures following the respective cold treatments (TP_2_,_4_). We quantified 58 primary metabolites, including 21 sugars/sugar phosphates/polyols, 13 organic acids, and 24 amino acids (including amines). A total of 95 lipid species were detected and measured in the spikes of both varieties, including 86 phospholipids such as PCs (phosphotidylcholine), PEs (phosphotidylethanolamine), PGs (phosphotidylglycerolipid), PIs (phosphoinositide), and lysophospholipids. In addition, we also quantified nine galactosyl lipids (galactosyldiaclyglycerols) in the spikes: digalactosyldiacylglycerols (DGDG), monogalactosyldiacylglycerols (MGDG), and sulfoquinovosyl diacylglycerols (SQDG).

### 3.2. Cold Treatment Affects Spike Metabolites in Wyalkatchem More than Young Spikes

**Figure 2:**
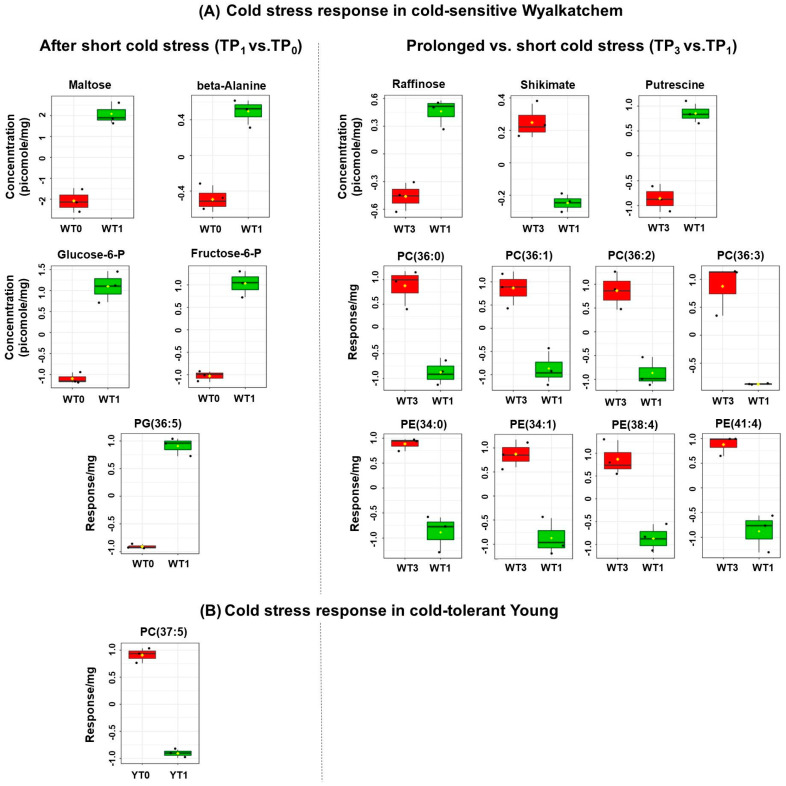
Pairwise comparisons of normalized and log_2_-transformed primary metabolites and lipids in the spikes of cold-sensitive Wyalkatchem (**A**) and cold-tolerant Young (**B**) after one night (TP_1_ vs. TP_0_) and prolonged (TP_3_ vs. TP_1_) of cold treatment. Metabolites and lipids shown are significantly different and the significance levels were determined using the Benjamini and Hochberg method [45] with a false discovery rate (FDR)-adjusted *p*-value of 0.05 as cut-off. There were three biological replicates (*n* = 3) for all the measured metabolites and lipids. W = Wyalkatchem; Y = Young

A short overnight cold treatment (≈12 h) revealed that four primary metabolites and one lipid were significantly affected in Wyalkatchem spikes: maltose (+17.8-fold), fructose-6-phosphate (+4.2-fold), glucose-6-phosphate (+4.7-fold), beta-alanine (+2.0-fold), and the lipid PG(36:5) (+1.2-fold) were all significantly increased (TP_1_ vs. TP_0_; Figure 2A). In contrast, the comparison of TP_1_ vs. TP_0_ in Young showed no significant changes in primary metabolites and only one lipid, i.e., PC (37:5), was significantly reduced (−16.5-fold; Figure 2B). The TP_2_ spike samples were harvested after 6 h of day-light and normal temperatures (21 °C) following the first night cold exposure (TP_1_). Comparison of TP_2_ to TP_1_ in Wyalkatchem spikes showed that only two metabolites were significantly changed: rather than induced at TP_1_, maltose was this time strongly repressed back to TP_0_ levels (−20-fold) and levels stay the same at TP_2_ and TP_4_, while 2-oxoglutarate was induced (+3.1-fold). There were no significant changes in the lipids at TP_2_ vs. TP_1_, indicating that the observed change in PG (36:5) at TP_1_ was maintained during day-time recovery (Supplementary Figures S1 and S2). In Young spikes, maltose was also repressed compared to TP_1_ (−10.7-fold) and we also found a significant increase in methionine levels at TP_2_ (+2.1-fold). There were no significant changes in the lipids, again indicating that the PC (37:5) lipid change at TP_1_ was maintained at TP_2_ (Supplementary Figures S1 and S2).

After a prolonged four-night cold treatment (TP_3_ vs. TP_1_ comparison), three primary metabolites changed significantly in Wyalkatchem spikes: raffinose (+1.9-fold), shikimate (−1.4-fold), and putrescine (+3.2-fold). Additionally, a series of saturated and unsaturated PC (36:x) lipids were significantly reduced at TP_3_ compared to TP_1_: PC (36:0) (−1.6-fold), PC (36:1) (−1.5-fold), PC (36:2) (−1.5-fold), PC (36:3) (−1.2-fold). Four PE lipid species were also significantly reduced: PE (34:0) (−1.4-fold), PE (34:1) (−1.4-fold), PE (38:4) (−1.2-fold), and PE (41:4) (−1.4-fold) (Figure 2A). In Young spikes, the prolonged cold treatment (TP_3_) did not cause any significant changes in primary metabolites and lipids compared to TP_1_ (Figure 2B). Comparing the prolonged cold treatment samples with their equivalent day-time samples (TP_4_ vs. TP_3_) showed the same significant reduction in maltose levels (−8.8-fold) as observed in Wyalkatchem spikes between TP_2_ and TP_1_, as well as an increase in quinate levels (+1.7-fold). There were no significant changes in lipid levels (Supplementary Figures S1 and S2). In Young spikes, the TP_4_ vs. TP_3_ pairwise comparison did not reveal any significant changes in primary metabolites or lipids (Supplementary Figures S1 and S2). These data indicate that the cold treatment has a more drastic effect on spikes of the cold-sensitive line Wyalkatchem compared to the tolerant line Young.

### 3.3. Varietal Differences in Metabolite and Lipid Levels before and after Cold Treatment

**Figure 3:**
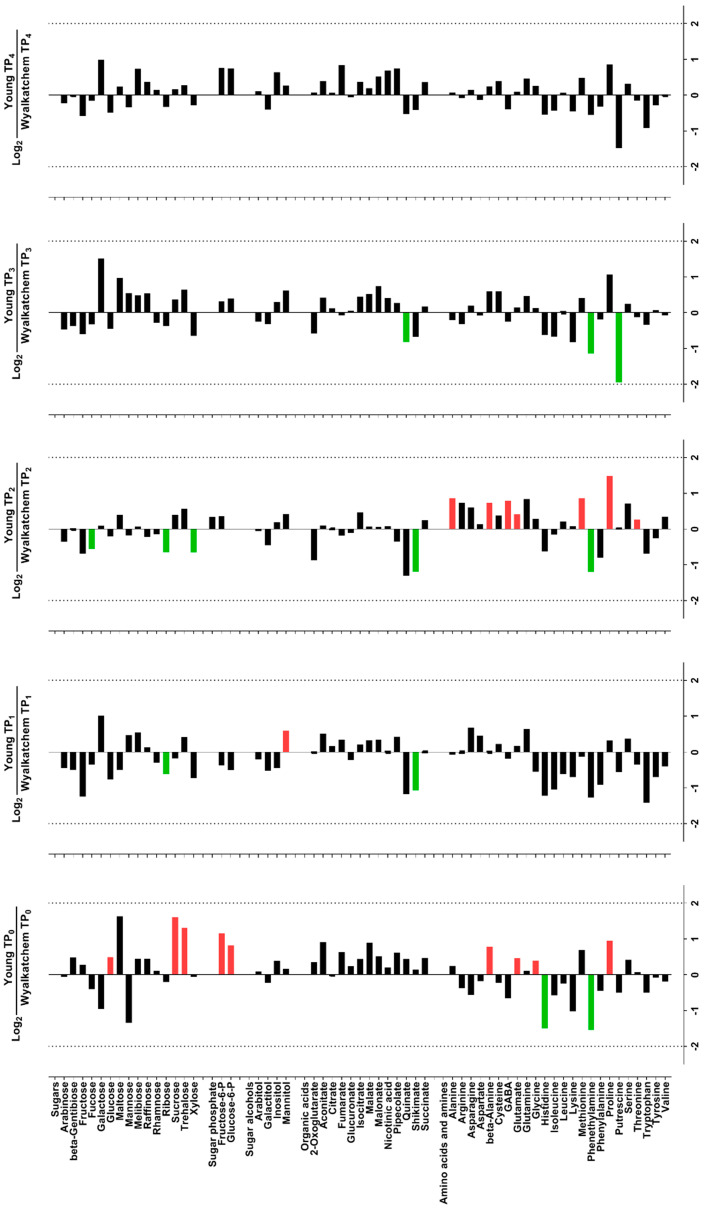
Log_2_-fold changes of primary metabolites in spikes of cold-tolerant Young (Y) compared to cold-sensitive Wyalkatchem (W) at each time point. Fold changes were calculated by dividing the concentration of Young by the concentration of Wyalkatchem at each time point, followed by log_2_-transformation. FDR-adjusted *p*-value of 0.05 was set as the cut-off. Green = significantly lower in Young, or higher in Wyalkatchem; red = significantly higher in Young, or lower in Wyalkatchem. *n* = 3.

**Figure 4:**
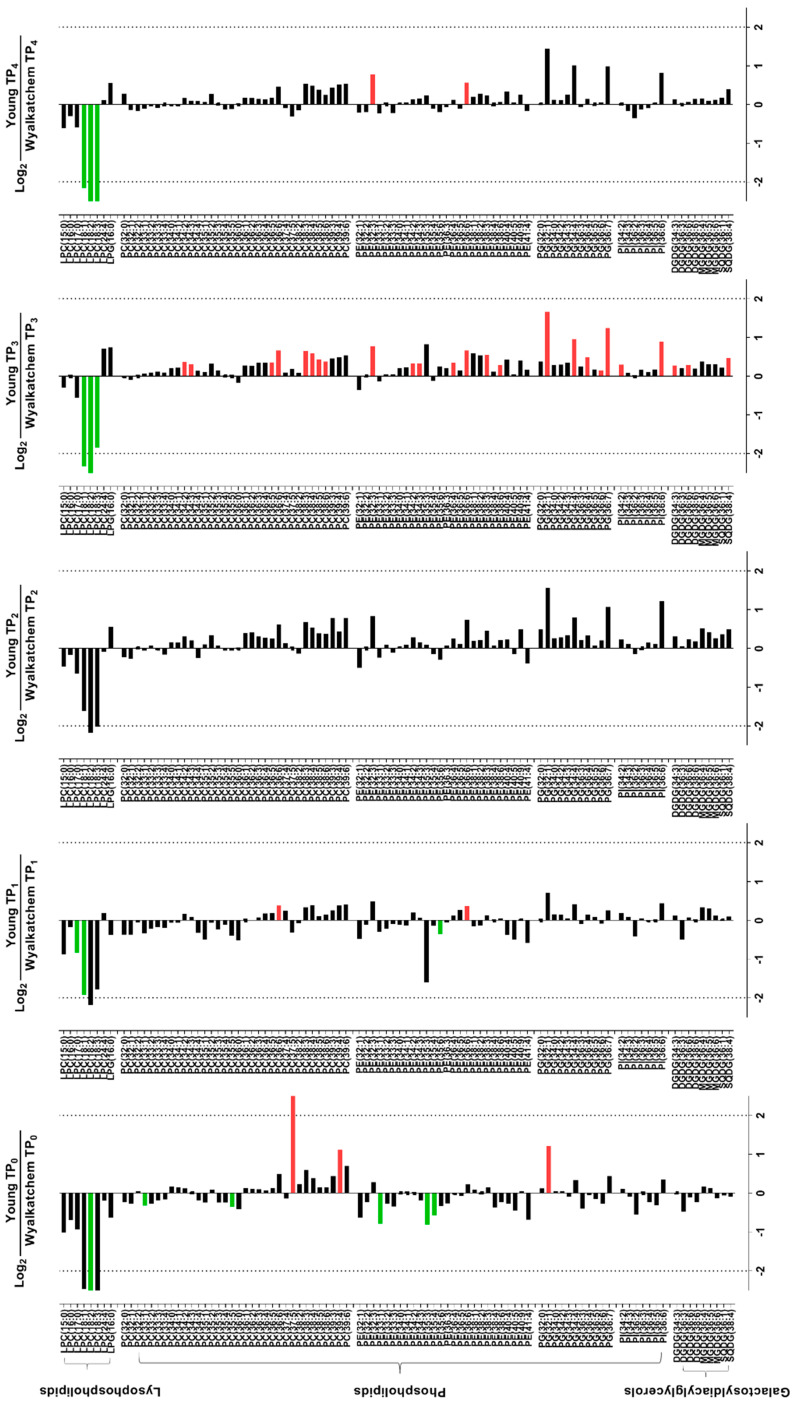
Log_2_-fold changes of lipids in the spikes of cold-tolerant Young (Y) compared to cold-sensitive Wyalkatchem (W) at each time point. Fold changes were calculated by dividing the concentration of Young by the concentration of Wyalkatchem at each time point, followed by log_2_-transformation. FDR-adjusted *p*-value of 0.05 was set as the cut-off. Green = significantly lower in Young, or higher in Wyalkatchem; red = significantly higher in Young, or lower in Wyalkatchem. *n* = 3.

**Figure 5:**
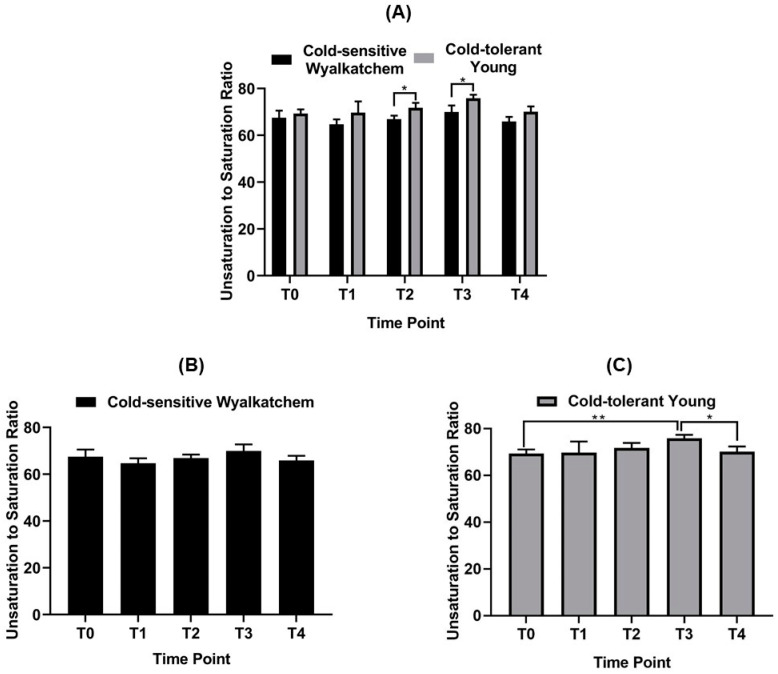
(**A**) Comparison of unsaturated to saturated lipid ratio between Wyalkatchem and Young across the time points. (**B**) Comparison of unsaturated to saturated lipid ratio in Wyalkatchem across the time points. (**C**) Comparison of unsaturated to saturated lipid ratio in Young across the time points. Student’s *t*-test was used to compare the ratio for a time point with its following time point. Error bars indicate the standard error mean (SEM) of three biological replicates (*n* = 3). Significantly different ratios were labelled with an asterisk (* = *p* < 0.05, ** = *p* < 0.01).

Before the plants were subjected to cold stress (TP_0_), there were already significant differences in the levels of primary metabolites and lipids in the spikes of cold-tolerant Young and cold-sensitive Wyalkatchem. Nine primary metabolites were significantly higher in Young compared to Wyalkatchem at TP_0_: glucose (+1.4-fold), sucrose (+3.1-fold), trehalose (+2.5-fold), fructose-6-phosphate (+2.2-fold), glucose-6-phosphate (+1.8-fold), beta-alanine (+1.7-fold), glutamate (+1.4-fold), glycine (+1.3-fold), and proline (+1.9-fold). Two metabolites were significantly lower in Young compared to Wyalkatchem spikes: histidine (−2.8-fold) and phenethylamine (−2.9-fold) (Figure 3). Three unsaturated lipid species were significantly higher at TP_0_ in Young spikes compared to Wyalkatchem: PC (37:5) (+12.9-fold), PC (39:4) (+2.2-fold), and PG (32:1) (+2.3-fold). In contrast, six unsaturated lipids were significantly lower at TP_0_ in Young spikes compared to Wyalkatchem: LPC (18:2) (−9.2-fold), PC (33:1) (−1.2-fold), PC (35:5) (−1.3-fold), PE (33:1) (−1.7-fold), PE (35:3) (−1.7-fold), and PE (35:4) (−1.5-fold) (Figure 4). Before the start of the cold treatment, there was no significant difference in the overall unsaturated to saturated lipid ratio between the two varieties (Figure 5A).

After one night of cold exposure (TP_1_), three metabolites were found to be significantly different between the two varieties. The osmolyte mannitol accumulated to significantly higher levels (+1.5-fold), while ribose (−1.5-fold) and shikimate (−2.1-fold) were significantly lower in Young compared to Wyalkatchem spikes (Figure 3). Two lysophosphatidylcholine lipids were significantly lower in Young spikes compared to Wyalkatchem: LPC (17:0) (−1.8-fold) and LPC (18:1) (−3.8-fold). Two lipids that contain two polyunsaturated acyl chains in their structures, PC (36:6) (+1.3-fold) and PE (36:6) (+1-3-fold), had significantly higher levels in Young spikes compared to Wyalkatchem at TP_1_ (Figure 4). However, again, at TP_1_ there was no significant difference in the overall ratio of unsaturated to saturated lipids between the two varieties (Figure 5A).

When comparing the TP_2_ day-time samples between the two varieties, seven metabolites were significantly higher in Young compared to Wyalkatchem spikes. All of them were either amino acids or amines: alanine (+1.7-fold), beta-alanine (+1.7-fold), GABA (+1.7-fold), glutamate (+1.3-fold), methionine (+1.8-fold), proline (+2.8-fold), and threonine (+1.2-fold). Three sugars (fucose, −1.5-fold; ribose, −1.6-fold; xylose, −1.6-fold), one organic acid (shikimate, −2.3-fold), and one amine (phenethylamine, −2.3-fold) were significantly reduced at TP_2_ in Young spikes compared to Wyalkatchem (Figure 3). There were no significant differences in the lipids between the two varieties at TP_2_, but for the first time, Young spikes showed a significantly higher ratio in unsaturated to saturated lipids compared to the ratio in Wyalkatchem spikes (Figure 5A).

After prolonged cold exposure (TP_3_), three metabolites were decreased significantly in Young spikes compared to Wyalkatchem: quinate (−1.8-fold), methionine (−2.2-fold), and putrescine (−3.8-fold) (Figure 3). The largest difference between both wheat varieties at TP_3_ was in the lipid levels. Three lysophosphatidylcholines with a carbon backbone of 18 were significantly lower in Young spikes compared to Wyalkatchem: LPC (18:1) (−5.0-fold), LPC (18:2) (−7.3-fold), and LPC (18:3) (−3.6-fold). Coincidently, 25 lipids with a higher carbon backbone length of 36 and a higher poly-unsaturation level were increased significantly in Young spikes compared to Wyalkatchem (Figure 4). Interestingly, 10 of these 25 lipid species were found to contain two polyunsaturated acyl chains in their structures: PC (36:5) (+1.3-fold), PC (36:6) (+1.6-fold), PC (38:5) (+1.3-fold), PC (38:6) (+1.3-fold), PE (36:6) (+1.6-fold), PE (38:6) (+1.2-fold), PG (36:6) (+1.1-fold), PG (36:7) (+2.4-fold), PI (36:6) (+1.9-fold), and DGDG (36:6) (+1.2-fold). This change in the lipid spectrum at TP_3_ resulted in Young spikes showing a significantly higher unsaturated to saturated lipid ratio compared to Wyalkatchem (Figure 5A).

Comparison between Young and Wyalkatchem at the last day-time sampling point (TP_4_) showed that there were no significant differences in primary metabolites between the two wheat varieties (Figure 3). In terms of lipids, three lysophosphatidylcholines were again significantly lower in Young compared to Wyalkatchem spikes: LPC (18:1) (−4.5-fold), LPC (18:2) (−7.6-fold), and LPC (18:3) (−5.9-fold). However, two unsaturated phosphatidylethanolamine lipids species, PE (32:3) (+1.7-fold) and PE (36:6) (+1.5-fold), were significantly higher in Young spikes compared to Wyalkatchem (Figure 4). At TP_4_, there was no significant difference in the unsaturated to saturated lipid ratio between the two varieties (Figure 5A).

### 3.4. Differences in Primary Metabolite Levels between Spikes and Flag Leaves of Wyalkatchem and Young

**Figure 6:**
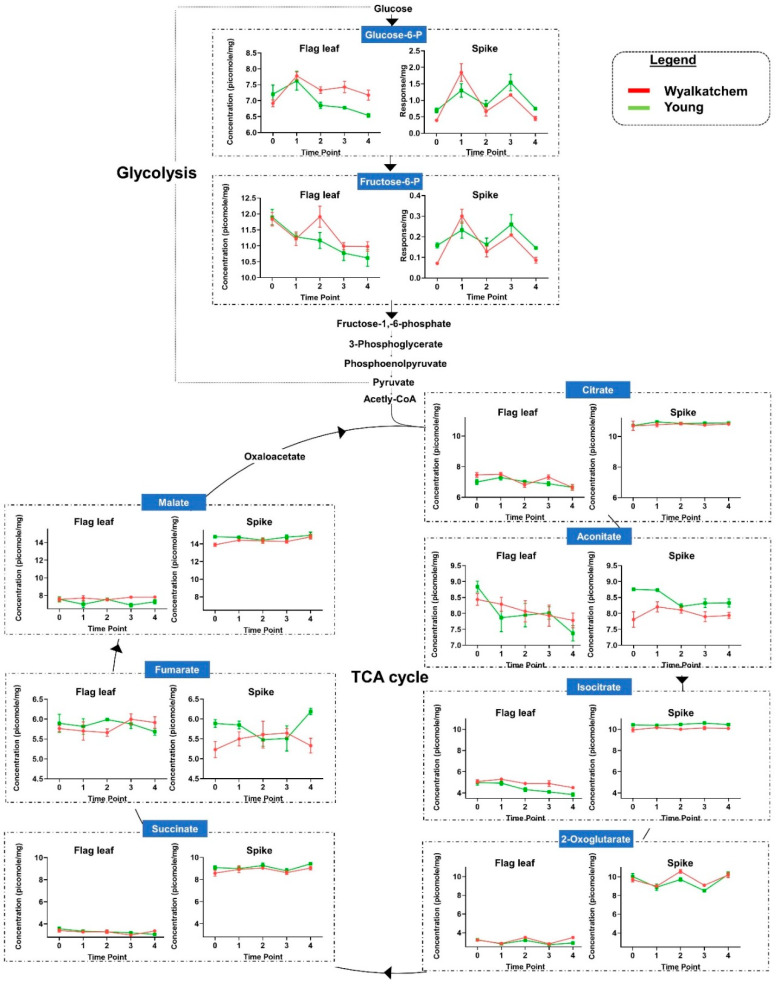
Comparison of primary metabolites involved in glycolysis and the tricarboxylic acid **cycle** (TCA) cycle, the two main energy-providing pathways of the cell. The graphs show differences in relevant primary metabolite concentrations (picomole/mg of fresh weight, Log_2_-transformed) between flag leaf and spike metabolite data. For glucose-6-phosphate and fructose-6-phosphate, the spike data compare the response normalized per mg of fresh weight. The graphs reveal differences in accumulation levels of these metabolites in cold-tolerant Young and cold-sensitive Wyalkatchem spikes. *n* = 4 ± SEM for flag leaf, *n* = 3 ± SEM for spike.

**Figure 7:**
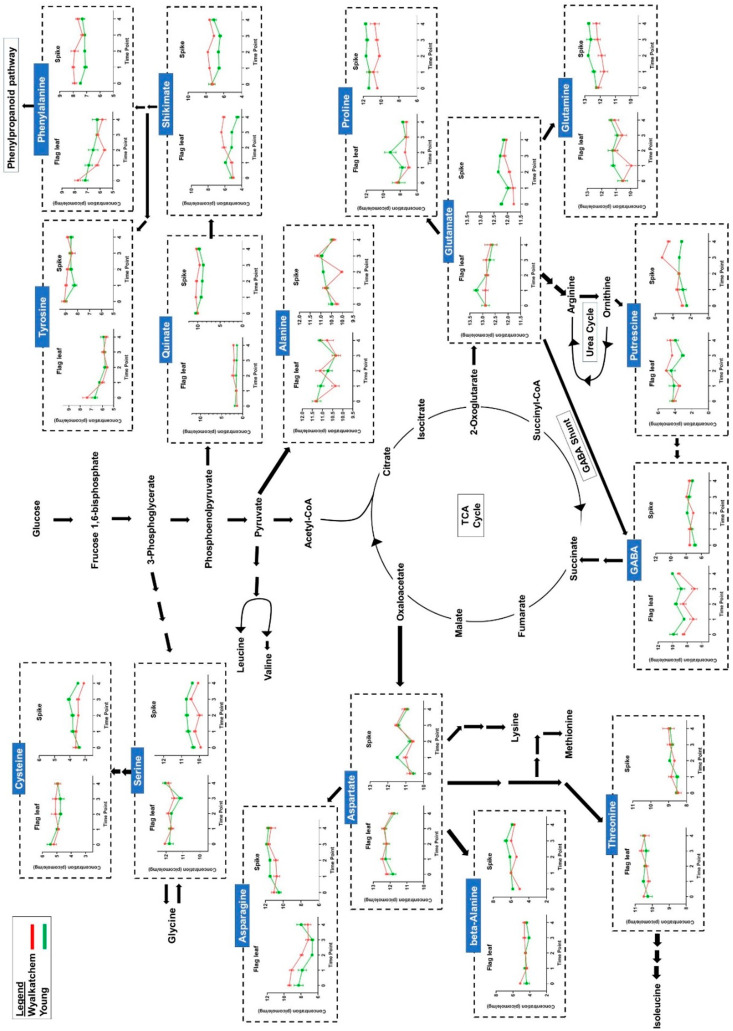
Comparison of amino acid and amine levels (picomole/mg of fresh weight and Log_2_-transformed), as well as their relationship to the main energy providing pathways (glycolysis and TCA cycle). The graphs show differences in metabolite levels between flag leaf and spike metabolite data, as well as differences in accumulation levels of those metabolites in cold-tolerant Young and cold-sensitive Wyalkatchem spikes. *n* = 4 ± SEM for flag leaf, *n* = 3 ± SEM for spike.

The spikes and flag leaves of the two wheat varieties showed significant differences in sugars and organic acids that are part of glycolysis and tricarboxylic acid (TCA) cycle. Twelve amino acids, putrescine, gamma-aminobutyric acid (GABA), and two organic acids (quinate and shikimate) that are involved in amino acid metabolism, showed significant differences between the spikes and flag leaves of the two wheat varieties (Figure 6 and Figure 7; for one-way ANOVA, see Supplementary Table S10). Of these 16 metabolites, eight were found to be significantly higher in the spikes compared to the flag leaves over the 4-day cold treatment: tyrosine, phenylalanine, quinate, shikimate, proline, glutamine, beta-alanine, and asparagine. Five amino acids (glutamate, aspartate, threonine, serine, and cysteine) and GABA were significantly lower in the spikes compared to the flag leaves. Putrescine was slightly lower in the spikes compared to the flag leaves, especially in the cold-tolerant variety Young. Interestingly, the response of alanine increased significantly from TP_1_ to TP_3_ in spikes, while it decreased for the same time points in flag leaves of both varieties. The results in Figure 6 and Figure 7 reveal that the overall response of metabolites to cold is quantitatively stronger in the reproductive tissues (spikes) compared to the flag leaves for both the sensitive and tolerant wheat line.

### 3.5. Differences in Metabolite and Lipid Levels between Spikes and Flag Leaves of Wyalkatchem and Young

**Figure 8:**
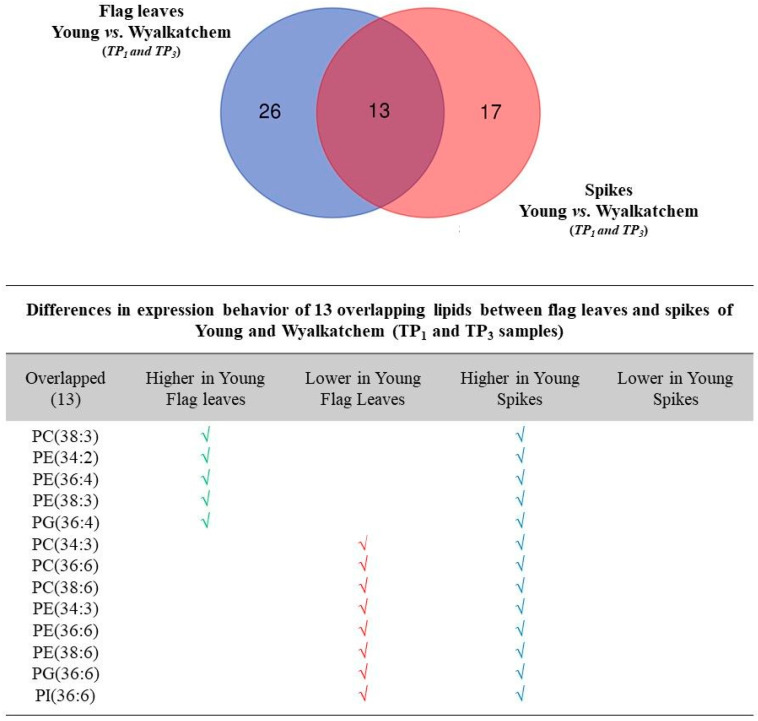
Differences in differentially expressed lipid species between flag leaves and spikes of cold-tolerant Young and cold-sensitive Wyalkatchem. The Venn diagrams show the degree of overlap between differentially expressed cold-induced lipid species in flag leaves and spikes for Young and Wyalkatchem. Thirteen lipid species were commonly expressed in flag leaves and spikes at TP_1_ and TP_3_. The table below the diagram shows how these 13 lipid species are expressed differently in flag leaves and spikes of Wyalkatchem and Young.

The most dramatic changes in primary metabolites and lipids occur at TP_1_ and TP_3_. We therefore compared the response at these time points between flag leaves and spikes for Young and Wyalkatchem. In terms of primary metabolites, cold treatment only affected 10 and six primary metabolites in flag leaves and spikes, respectively, and only three of these metabolites were common to both tissues: putrescine, quinate, and shikimate (Supplementary Figure S3). Interestingly, all three of these overlapping metabolites were lower in both Young flag leaves and spikes compared to Wyalkatchem. We observed a larger number of changes in lipid composition for both wheat varieties at TP_1_ and TP_3_ (Figure 8). The results show that there were both similarities and differences in lipid accumulation between flag leaves and spikes (Figure 8). Thirteen lipids were found to overlap between the lipid species of flag leaves and spikes and all of them were polyunsaturated lipids. Of the common lipids, five were accumulating to higher levels in both spike and flag leaf tissues of cold-tolerant Young compared to cold-sensitive Wyalkatchem: PC (38:3), PE (34:2), PE (36:4), PE (38:3), and PG (36:4). Eight unsaturated lipids were found to be lower in Young flag leaves when compared to Wyalkatchem, but their levels were higher in Young spikes compared to Wyalkatchem spikes: PC (34:4), PC (36:6), PC (38:6), PE (34:3), PE (36:6), PE (38:6), PG (36:6), and PI (36:6). Interestingly, levels of all these 13 polyunsaturated lipid species showed quantitatively higher levels in Young compared to Wyalkatchem spikes (Figure 8). These results indicate that there are quantitative and qualitative differences in cold acclimation between sensitive variety Wyalkatchem and tolerant Young.

## 4. Discussion

### 4.1. Are Wheat Spikes or Flag Leaves Better for Cold Tolerance Phenotyping?

We have previously compared the effect of cold stress on metabolite levels during cold acclimation in flag leaves of cold-tolerant Young and cold-sensitive Wyalkatchem [30]. For the ultimate aim of phenotyping, flag leaves are easy to collect, especially in the field. The motivation to also study metabolite changes in wheat spikes during cold acclimation came from the fact that spikes are the organs of the wheat plant where grain yield is determined. Spike material at the stage of highest sensitivity to cold, the YM stage of pollen development [30], is harder to collect. However, this tissue may provide metabolite information that is more directly correlated with the cold tolerance/sensitivity mechanism. If we can prove that the metabolite changes in flag leaves and spikes show significant similarities, then we can be confident that flag leaf material suits our phenotyping needs.

An important difference between flag leaf and YM stage spike tissues is that the former is a static photosynthetic source tissue, whilst the latter is a young and actively growing tissue in need of sugar supply to drive reproductive development (sink tissue). YM stage spikes are shielded from daylight by the surrounding leaf sheaths and have a light-green appearance; there may be a low amount of chlorophyll and photosynthetically active chloroplasts in cells of the outside tissue layers of the florets (glumes, lemma, and awns), but the anthers that produce pollen inside the florets are yellow and at the YM stage are non-photosynthetic sink tissues [38,39]. Sink strength is defined as the competitive ability of an organ to attract and store assimilates [46]. At the reproductive stage, developing wheat spikes need a strong sink strength to attract sugars for reproductive development and grain filling [32,33,34,36,47]. In rice and wheat, cold and drought stress reduce sink strength in lines that are more sensitive to those stresses and stress tolerance correlates with a higher capability to maintain sink strength [38,48]. The higher sugar levels in Young compared to Wyalkatchem spikes before cold exposure therefore indicate that Young spikes have a higher sink strength than Wyalkatchem spikes.

The spikes of the cold-tolerant variety Young did not show any significant primary metabolite changes upon short or prolonged exposure to cold, while cold-sensitive Wyalkatchem did show some metabolites that were significantly affected by cold. In the flag leaves there were more changes in primary metabolites in response to the same cold treatment [30]. It is possible that the cold treatment at the spike level is somewhat less stringent compared to the flag leaf. While the flag leaf is directly exposed to cold air, at the YM stage the spike is still protected inside the leaf sheath of the flag leaf and the penultimate leaf. However, spikes of both wheat varieties show a clear lipid response to cold, which is similar to that of the flag leaf [30]. This indicates that the YM stage spikes have experienced cold stress. Alternatively, they responded to a cold stress signal coming from the vegetative plant parts.

Most metabolites that were detected are present at higher levels in actively growing spikes than in flag leaves and are therefore easier to detect in spike tissues. Despite quantitative and qualitative differences in the cold acclimation response between spike and flag leaves, the message we obtained in terms of metabolite profiles and differences in cold acclimation between the tolerant and sensitive wheat line is quite similar. This observation also strengthens the conclusion that the response we observed is a true defensive acclimation response to the chilling/freezing treatment we imposed.

### 4.2. Spikes of Cold-Tolerant Young Are Potentially Better Prepared for Low Temperature Stress

Levels of important sugars (glucose, sucrose, trehalose, fructose-6-phosphate, and glucose-6-phosphate) were significantly higher under unstressed conditions (TP_0_) in spikes of cold-tolerant Young compared to cold-sensitive Wyalkatchem. Higher levels of these sugars may indicate that Young spikes have a higher sink strength and are more metabolically active than Wyalkatchem spikes under normal growth conditions. High sugar levels also play a role in osmotic adjustment and membrane protection during water and cold stress [49]. In addition, proline and β-alanine levels were also significantly higher in Young spikes before cold stress treatment. Proline is an osmolyte with protective functions in plant tissues under a variety of stresses (drought, salinity, cold, and heat) [50,51,52,53,54]. β-Alanine in spikes of the sensitive variety Wyalkatchem is only increased after the first night of cold exposure. β-Alanine is a non-proteinogenic amino acid that is increased during abiotic stress conditions [50,55,56]; it is a coenzyme A precursor that is involved in the tricarboxylic acid cycle, as well as phospholipid and fatty acid metabolism [57]. β-Alanine serves also as a substrate for β-alanine betaine synthesis, a quaternary ammonium compound with osmo-protective functions under stress conditions [58,59,60,61]. The polyol sugar mannitol accumulates to higher levels in Young spikes after the first cold night (TP_1_). Mannitol acts as a radical scavenger to protect against ROS under abiotic stress conditions [62,63,64]. The flag leaf study showed similar observations [30].

Some other sugars with protective functions are mainly accumulating in spikes of the sensitive line Wyalkatchem. Maltose levels increase after the first night of cold stress exposure, but in Young the increase in maltose remained insignificant. Maltose is a transitory starch breakdown product and this breakdown is induced during the day under photorespiratory conditions. Maltose metabolism is regulated by the circadian clock, day-length, and temperature [65]. Maltose plays a role in stabilizing membranes and proteins [66,67]. In Arabidopsis, maltose functions as a compatible solute and a stabilizing factor in the chloroplast stroma during temperature stress [55]. In vitro assays indicate that maltose protects proteins, membranes, and the photosynthetic electron transport chain during freezing stress. Increased maltose levels under cold stress could be caused by increased expression of β-amylase [55,65].

Spikes of the cold-sensitive variety Wyalkatchem also showed increased levels of putrescine and raffinose after prolonged cold stress (TP_3_). Putrescine is a polyamine and amino acid breakdown product with growth-regulatory properties that plays a role in abiotic stresses [68,69]. Raffinose is a soluble galactosyl-sucrose carbohydrate that plays a role in stabilizing photosystem II during freeze-thaw cycles in Arabidopsis [70]. Raffinose acts as an antioxidant that can protect cells from oxidative stress damage by scavenging ROS [62,63,64,71,72].

In contrast to Young, where a protection mechanism involving higher sink strength and accumulation of osmolytes (sugars, amino acids) appears to be constitutively present, Wyalkatchem activates primary metabolite changes after the first night and during prolonged cold exposure. Spikes of cold-tolerant Young may therefore be better prepared against cold stress from the start. This knowledge can be exploited in phenotyping approaches for cold tolerance. The primary metabolite response in cold-sensitive Wyalkatchem is a cold stress response. Being a sensitive variety, Wyalkatchem responds with osmolyte synthesis, possibly to control water loss caused by membrane damage, and with activation of a signature oxidative stress and ROS protection mechanism. The same type of response was also observed in Wyalkatchem flag leaves. Osmolytes can therefore be used as indicator for the secondary water stress response caused by cold-induced membrane damage [30,73].

### 4.3. Lipid Re-Modelling Is Critical for Cold Acclimation in Wheat Spikes

Modifying membrane lipid composition forms an important part of cold acclimation. Similar to what we found for flag leaves, in wheat spikes chilling stress had a more drastic effect on lipids compared to primary metabolites. Under unstressed conditions, Young spikes showed higher levels of three unsaturated phospholipids, with PC (37:5) being the most prevalent one. Unstressed Wyalkatchem spikes had higher levels of five different unsaturated phospholipids. Interestingly, in Young spikes the high level of PC (37:5) was reduced drastically after overnight cold stress. PC (37:5) was much lower in unstressed Wyalkatchem spikes, nor was the level of this lipid strongly repressed by overnight cold treatment. Instead, Wyalkatchem increased PG (36:5) levels upon cold exposure. PG (32:1) is higher in Young compared to Wyalkatchem at the unstressed and prolonged cold stress stage (TP_3_). PG lipids are the main phospholipid class in thylakoid membranes of higher plants [74]. Plant cells contain proplastids that can multiply and develop into specialised plastids such as chloroplasts. Plants increase the degree of fatty acid unsaturation and the content of phospholipids when they are exposed to low, non-freezing temperatures [75,76]. PG lipids contain trans-Δ3-hexadecenoic acid (C16:1t), a monounsaturated 16-carbon fatty acid with a trans-double bond at position C-3); they are major fatty acids involved in decreasing the phase transition temperature of thylakoid PG and maintaining membrane fluidity at lower temperatures [74,77,78,79,80]. The highly unsaturated PG (36:5) lipid in Wyalkatchem spikes consists of 18:2 and 18:3 acyl chains and may protect the plastidial/thylakoid membranes. Interestingly, this species was also found to increase significantly in the flag leaves of the same variety after overnight cold stress (TP_1_) [30].

After prolonged cold stress, a series of PC (36:x, with x ranging from 0 to 3) lipids, as well as several saturated and polyunsaturated PE lipids, were decreased in cold-sensitive Wyalkatchem and not in cold-tolerant Young spikes. This may be a consequence of their utilization as substrates to produce PAs by phospholipases to trigger a further response against cold stress. Alternatively, the decrease in those PC and PE lipids in Wyalkatchem could be the consequence of membrane damage and/or lipid degradation.

In Young spikes, the first cold night increased the levels of the polyunsaturated lipids PC (36:6) and PE (36:6). The result is a net increase in the degree of fatty acid unsaturation and phospholipid content in Young spikes. In Arabidopsis, PC lipids are amongst a group of lipids that increased during cold acclimation [81]. The higher lipid unsaturation to saturation ratio in Young compared to Wyalkatchem improves the ability to maintain membrane fluidity at low temperatures.

Under unstressed conditions, and particularly after prolonged exposure to cold, most short-chain lysophospholipids (LPC) were higher in Wyalkatchem spikes compared to Young. PCs and PEs are the most abundant plasma membrane lipids in plants and animals [30]. Plants can lower the ratio of PC to PE during cold stress via a hydrolysis reaction catalyzed by phospholipase D (PLDα). Hydrolysis of PC lipids at the sn-1 or sn-2 positions of the glycerol backbone results in formation of LPC and phosphatidic acid (PA), a signaling molecule involved in cold and ABA signaling [82,83,84,85,86,87,88,89,90]. LPC levels are generally higher in Wyalkatchem spikes at all time points, but most obviously after prolonged cold treatment (TP_3,4_). This was not observed in flag leaves, where none of the LPC lipids were significantly different between Wyalkatchem and Young. In Young spikes, cold-induced PC (37:5) catabolism does not seem to impact LPC levels. PC (39:4) is also higher in unstressed Young spikes; this lipid does not seem to be reduced as dramatically as PC (37:5), but is also induced in Wyalkatchem to a level similar to Young after overnight cold exposure. Both PC (37:5) and PC (39:4) can serve as substrates for PLDα to produce the cold response signaling molecule PA [87,88], so Young could initially benefit from higher levels of these lipids to produce PA and trigger a cold response quicker and more efficiently than Wyalkatchem. Higher levels of LPC (18:1), LPC (18:2) and LPC (18:3) in Wyalkatchem spikes after prolonged cold stress (TP_3_ and TP_4_) may indicate that prolonged cold stress has caused degradation of PC lipids, causing damage to the plasma membrane. This may then lead to water loss and electrolyte leakage.

Maintenance of plastid functionality during chilling conditions also requires changes in membrane lipid composition. The wheat spike consists of several tissue types (glumes, lemma, awns, anthers, and ovule), most of which will become photosynthetically active when the spike extrudes from the leaf sheath of the flag leaf later on in development. As mentioned earlier, plant cells contain proplastids that can multiply and develop into specialized plastids such as chloroplasts. Light exposure leads to synthesis of photosynthetically active thylakoid membranes and differentiation into chloroplasts [91,92,93]. The thylakoid membrane of chloroplasts is composed mainly of galactosylglycerolipids, consisting of 50% MGDG, 26% DGDG, and 24% SQDG lipids, as well as other galactosylglycerolipid species and PG lipids [94]. Low temperature acclimation can lead to an increased proportion of highly unsaturated fatty acids in galactolipids, such as linolenic acid (C18:3) [95]. Levels of DGDG (34:3), DGDG (36:6), and SQDG (38:4) in Young spikes after prolonged cold treatment (TP_3_) may help this cold-tolerant variety to maintain better chloroplast functionality. Higher levels of DGDG improves ionic permeability of the chloroplasts and preserves activity of membrane proteins [96,97]. Experiments using desaturase enzyme mutants in Arabidopsis have illustrated the importance of glycerolipid composition and the degree of fatty acid desaturation in controlling membrane performance during cold stress [77].

## 5. Conclusions

Profiling of primary metabolite and lipid changes in response to cold treatment of wheat spikes provided a better understanding about differences in cold acclimation of cold-tolerant Young and cold-sensitive Wyalkatchem. The changes in metabolomes and lipidomes of spikes were similar to those of flag leaves in many ways [30], but there were clearly some interesting quantitative and qualitative differences in accumulation of osmolytes, ROS protective compounds, and lipids. This means that both flag leaf and spike metabolite and lipidome analysis are complementary in terms of physiological information, and they can be used interchangeably for cold tolerance phenotyping in wheat. Flag leaf tissue is easier to collect, while spike tissue offers an advantage in terms of detectability because of the presence of higher levels of some of the metabolites. Both the flag leaf and spike datasets pointed out that re-modelling of membrane lipids is critical for adaptation to low temperature stress. Controlling the unsaturated lipid levels to maintain membrane fluidity at low temperatures appears to be a distinguishing factor between cold-tolerant line Young and cold-sensitive Wyalkatchem. However, the change in the ratio of saturated to unsaturated lipids is small and there are also important changes in the saturated lipid spectrum. We do not know the biochemical structure of the lipid species that are affected by cold, nor do we know their exact role in controlling membrane functionality or which cellular membranes they are associated with. We identified some individual lipid species that stand out in the response of the cold-tolerant and sensitive wheat line, but for phenotyping purposes we will at this stage observe changes in the entire lipid spectrum. Experiments are in progress to demonstrate whether lipid profiling proves to be reliable in a wider variety of cold-tolerant and sensitive wheat lines and whether the changes in lipid spectrum matches the ones observed in Young and Wyalkatchem. When lipid profiling proves to be reliable, we can deploy the technology in QTL mapping or genome-wide association studies (GWAS) to identify to identify genetic markers that can be used to predict the cold tolerance phenotype. In addition, the data presented here have provided new avenues to investigate genes involved in metabolic and lipid biochemical processes using transcriptome analysis. Combination of metabolome and transcriptome analysis can further improve our understanding of how cold tolerance is controlled in wheat.

## Supplementary Materials

The following supplementary data files are available online at https://www.mdpi.com/2073-4409/9/5/1309/s1, Figure S1: log_2_-fold changes of primary metabolite concentrations in the spikes of cold-sensitive Wyalkatchem (W) and cold-tolerant Young (Y) after one night (TP_1_ vs. TP_0_) and prolonged (TP_3_ vs. TP_1_) exposure to cold, and the day-time recoveries after each of these cold treatments (TP_2_ vs. TP_1_ and TP_4_ vs. TP_1_), Figure S2: log_2_-fold changes of lipid concentrations in the spikes of the cold-sensitive Wyalkatchem (W) and cold-tolerant Young (Y) after one night (TP_1_ vs. TP_0_) and prolonged (TP_3_ vs. TP_1_) exposure to cold, and the day-time recoveries after each of these cold treatments (TP_2_ vs. TP_1_ and TP_4_ vs. TP_3_), Figure S3: Differences in differentially expressed primary metabolites between flag leaves and spikes of cold-tolerant Young and cold-sensitive Wyalkatchem. The Venn diagrams show the degree of overlap between differentially expressed cold-induced metabolites in flag leaves and spikes for Young and Wyalkatchem. Three metabolites were commonly expressed in flag leaves and spikes at TP_1_ and TP_3_. The table below shows how these three metabolites are expressed differently in Wyalkatchem and Young flag leaves and spikes, Table S1: Multiple reaction monitoring (MRMs) established for authentic sugars, organic acids, amino acids, and amines used for identification and quantification of these compounds in this study, Table S2: Multiple reaction monitoring (MRMs) established for the seven external authentic lipids, and 95 phospholipids used for identification of these compounds in this study, Table S3: Concentrations of primary metabolites (sugars, organic acids, amino acids, and amines) measured in Wyalkatchem and Young spikes across all time points, Table S4: Normalized lipid responses for the measured lipids in the Wyalkatchem and Young spikes across all the time points, Table S5: Fold changes ± SEM for the pairwise comparisons of metabolites in Young and Wyalkatchem spikes, Table S6: Fold changes ± SEM for the pairwise comparisons of lipids in Young and Wyalkatchem spikes, Table S7: Multiple comparisons of primary metabolites in Wyalkatchem, Young, Wyalkatchem vs. Young across all time points using one-way ANOVA (with FDR-adjusted *p* < 0.05), followed by Tukey test to determine which specific two groups are significantly different, Table S8: Multiple comparisons of phospholipids in Wyalkatchem, Young, Wyalkatchem vs. Young across all time points using one-way ANOVA (with FDR-adjusted *p* < 0.05), followed by Tukey test to determine which specific two groups are significantly different, Table S9: Changes in the unsaturated to saturated lipid ratio in Wyalkatchem and Young during the time-course experiment, Table S10: One-way ANOVA analysis and Tukey test for comparison of primary metabolite levels between flag leaves and spikes in Wyalkatchem and Young.
