# Supplementary material for: Phenotyping the Chilling and Freezing Responses of Young Microspore Stage Wheat Spikes Using Targeted Metabolome and Lipidome Profiling
Source: Cells. 2020 May 25;9(5):1309. doi: 10.3390/cells9051309 (PMC7291281; doi:10.3390/cells9051309)
Supplement: Supplementary file 1 [file cells-09-01309-s001.zip › Supplement/Figure S1-S3.pdf]

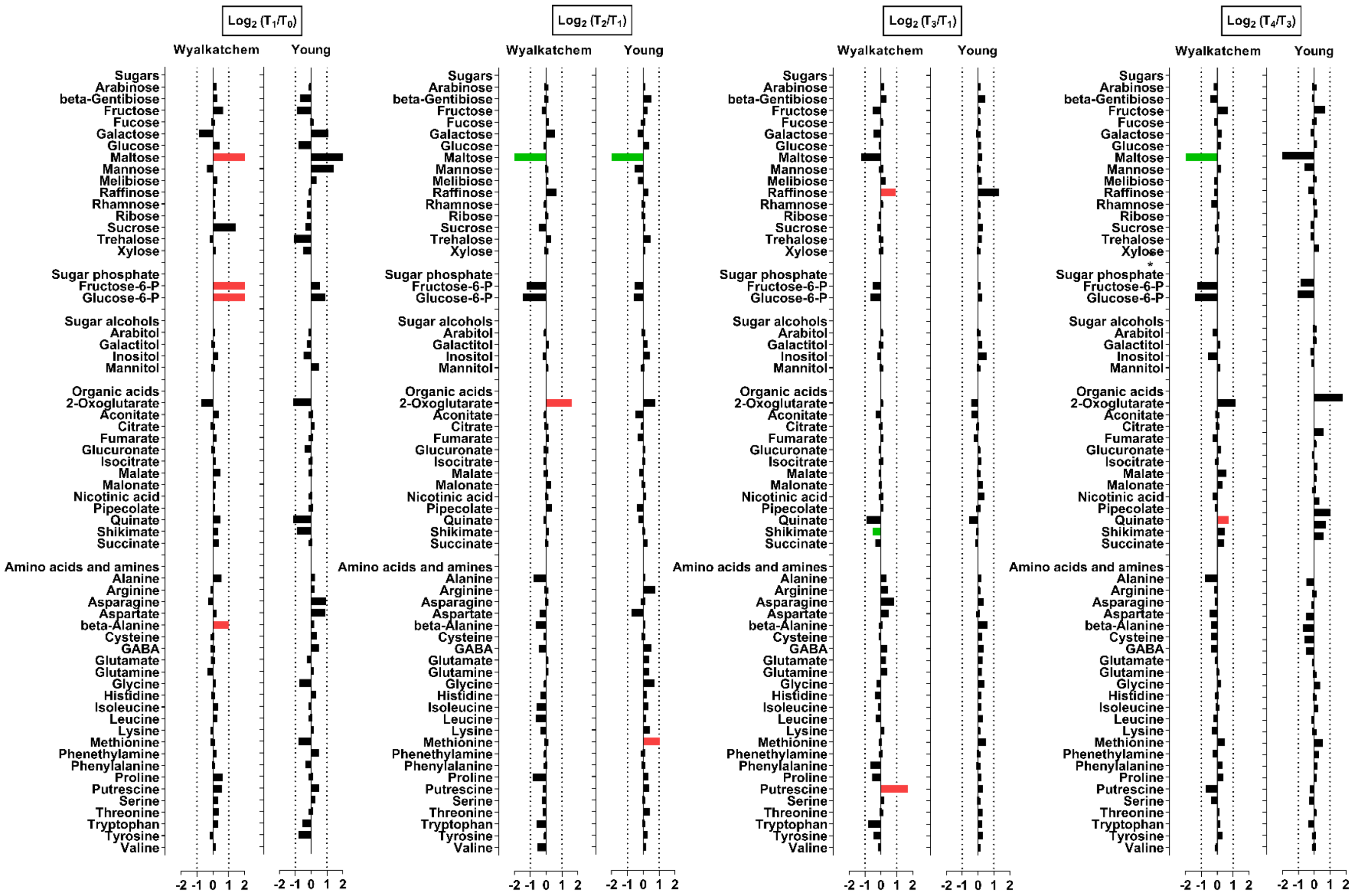

Figure S1. Log_2-fold changes of primary metabolite concentrations in the spikes of cold-sensitive Wyalkatchem (W) and cold-tolerant Young (Y) after one night (TP_1 vs. TP_0) and prolonged (TP_3 vs. TP_1) exposure to cold, and the day-time recoveries after each of these cold treatments (TP_2 vs. TP_1 and TP_4 vs. TP_1). n =3.

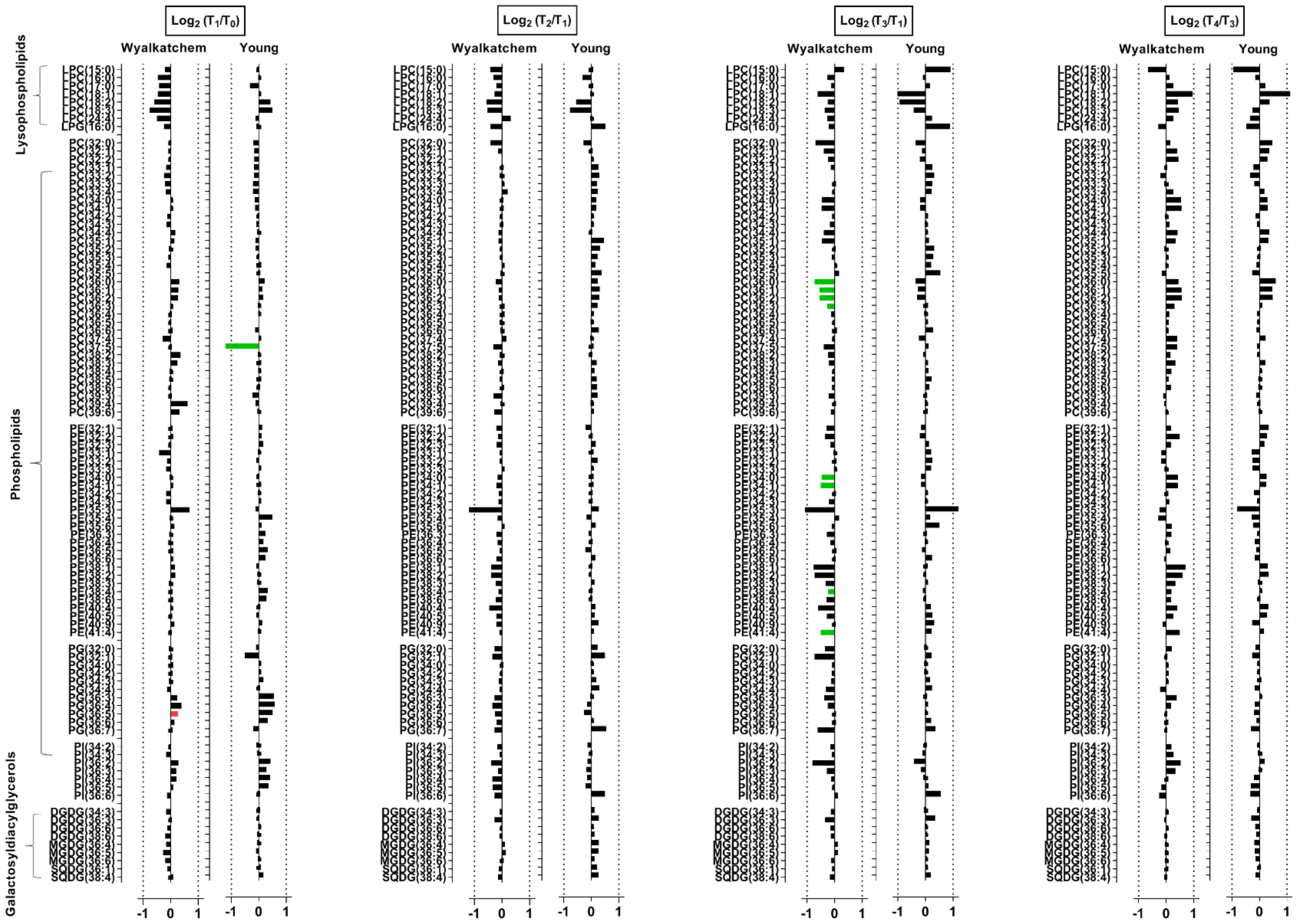

Figure S2. Log_2-fold changes of lipid concentrations in the spikes of the cold-sensitive Wyalkatchem (W) and cold-tolerant Young (Y) after one night (TP_1 vs. TP_0) and prolonged (TP_3 vs. TP_1) exposure to cold, and the day-time recoveries after each of these cold treatments (TP_2 vs. TP_1 and TP_4 vs. TP_3). n = 3.

N

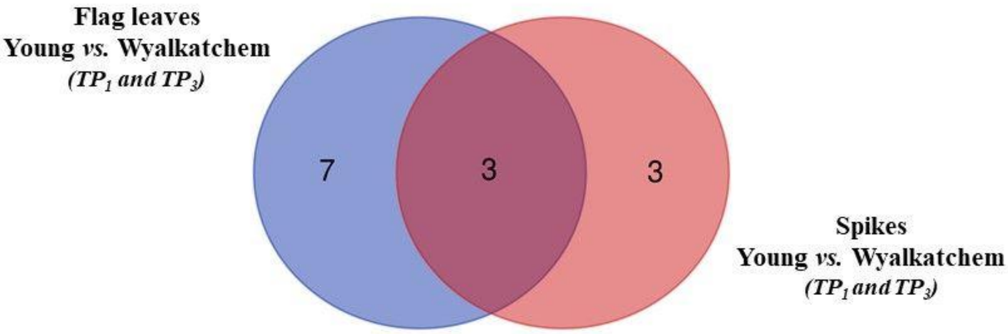

Differences in expression behavior of three overlapping metabolites between flag leaves and spikes of Young and Wyalkatchem (TP_1 and TP_3 samples)

Overlapped (3)	Higher in Young Flag leaves	Lower in Young Flag Leaves	Higher in Young Spikes	Lower in Young Spikes
Putrescine		√		√
Quinate		√		√
Shikimate		√		√

Figure S3. Differences in differentially expressed primary metabolites between flag leaves and spikes of cold-tolerant Young and cold-sensitive Wyalkatchem. The Venn diagrams show the degree of overlap between differentially expressed cold-induced metabolites in flag leaves and spikes for Young and Wyalkatchem. Three metabolites were commonly expressed in flag leaves and spikes at TP_1 and TP_3. The table below shows how these three metabolites are expressed differently in Wyalkatchem and Young flag leaves and spikes.
